# Test of Colonisation Scenarios Reveals Complex Invasion History of the Red Tomato Spider Mite *Tetranychus evansi*

**DOI:** 10.1371/journal.pone.0035601

**Published:** 2012-04-23

**Authors:** Angham Boubou, Alain Migeon, George K. Roderick, Philippe Auger, Jean-Marie Cornuet, Sara Magalhães, Maria Navajas

**Affiliations:** 1 INRA, UMR CBGP (INRA/IRD/Cirad/Montpellier SupAgro), CS 30016, Montferrier-sur-Lez, France; 2 Environmental Science Policy and Management, University of California, Berkeley, California, United States of America; 3 Faculdade de Ciências, Centro de Biologia Ambiental, Universidade de Lisboa, Lisbon, Portugal; Ghent University, Belgium

## Abstract

The spider mite *Tetranychus evansi* is an emerging pest of solanaceous crops worldwide. Like many other emerging pests, its small size, confusing taxonomy, complex history of associations with humans, and propensity to start new populations from small inocula, make the study of its invasion biology difficult. Here, we use recent developments in Approximate Bayesian Computation (ABC) and variation in multi-locus genetic markers to reconstruct the complex historical demography of this cryptic invasive pest. By distinguishing among multiple pathways and timing of introductions, we find evidence for the “bridgehead effect”, in which one invasion serves as source for subsequent invasions. *Tetranychus evansi* populations in Europe and Africa resulted from at least three independent introductions from South America and involved mites from two distinct sources in Brazil, corresponding to highly divergent mitochondrial DNA lineages. Mites from southwest Brazil (BR-SW) colonized the African continent, and from there Europe through two pathways in a “bridgehead” type pattern. One pathway resulted in a widespread invasion, not only to Europe, but also to other regions in Africa, southern Europe and eastern Asia. The second pathway involved the mixture with a second introduction from BR-SW leading to an admixed population in southern Spain. Admixture was also detected between invasive populations in Portugal. A third introduction from the Brazilian Atlantic region resulted in only a limited invasion in Europe. This study illustrates that ABC methods can provide insights into, and distinguish among, complex invasion scenarios. These processes are critical not only in understanding the biology of invasions, but also in refining management strategies for invasive species. For example, while reported observations of the mite and outbreaks in the invaded areas were largely consistent with estimates of geographical expansion from the ABC approach, historical observations failed to recognize the complex pathways involved and the corresponding effects on genetic diversity.

## Introduction

Specimen collection records are typically the only source of information concerning the historical distribution of a species. However, geographical range is not a static attribute of a species, a point particularly well illustrated in the case of invasive species, for which rapid range expansion can occur when individuals move from their native areas. Such dispersal events at large spatial scales and multiple colonisations, may occur within short time frames, making it difficult to reconstruct the history of accidentally introduced populations. In addition, introductions are typically characterized, at least initially, by small population sizes, often with no immediately recognizable environmental or agricultural impacts. Thus, initial stages of invasions often go undetected, further complicating historical reconstructions. Detecting minute arthropod pests, including spider mites, is particularly challenging. As a result high-impact invaders can reach unmanageable population numbers before major agricultural damage appears.

Most spider mites are small (adult females are <0.5 mm in size) and cryptic and easily transported hidden within commodities [1]. Yet, a single mated female may initiate a successful invasion due to a high reproductive rate [2], [3], [4]. Further, relevant historical records for mites are scarce and often incomplete and taxonomically misleading. For such species, understanding patterns of invasion requires rigorous methods that can go beyond occurrence records to distinguish among alternative invasion scenarios [5]. While genetic data have been used for some time to examine population structure, recent advances in both computer power and computational approaches, such as Approximate Bayesian Computation (ABC), make it possible to distinguish statistically among alternative invasion scenarios [6], [7], [8], [9], [10]. In the case of plant pests, knowing pathways and modalities of introduction are crucial for managers in developing policies to prevent damage and spread [11].

The red tomato spider mite, *Tetranychus evansi*, is native to South America [12], [13]. In the past half century it has expanded its range to North America, Africa and the Mediterranean basin, as well as far-east Asia and many tropical islands worldwide (see [14], [15] for a complete and chronological list). While the first collection records in the neotropics outside its native range are from Mauritius [16], [17], the mite was probably already present, but undetected, in other locations. For example, when serious outbreaks occurred on tomato in Kenya late 1990s, *T. evansi* was misidentified and confused with the closely related species *T. urticae*, until molecular markers were first applied to distinguish between the two species [18]. In fact, misidentification of spider mites and other small arthropod pests is a problem that frequently hampers research and control [19].

In a previous study of *T. evansi* based on mitochondrial (mt) Cytochrome Oxidase I (*COI*) and nuclear ribosomal Internal Transcribed Spacer (ITS) sequences we showed that multiple cryptic introduction events occurred in the Mediterranean basin corresponding to at least two main distinct lineages (I and II), each originating from distant geographical regions in South America [13]. Lineage I is more widely distributed and has colonized the entire Mediterranean basin, together with Africa and eastern Asia, causing serious outbreaks on solanaceous crops in southeast Africa and the Mediterranean region. Multiple introductions have the potential to obscure invasion history, but understanding such histories is important because multiple introductions may provide additional genetic variation necessary for invasion success [20], [21], [22], [23]. In addition, new invasions may derive from other invasive populations elsewhere, rather than directly from the native origin of the species [24], [25], [26], [27]. In the case of *T. evansi* the pathways and modes of colonization remain unresolved. Like many cryptic invaders, the colonization of new continents by *T. evansi* is possibly the result of several alternative invasive scenarios, each of which requires formal testing to provide an adequate spatial and temporal picture of the global invasion.

The development of multi-locus genetic markers (e.g. microsatellites), and the application of novel ABC methods can provide detailed estimates of demographic and historical parameters allowing quantitative comparisons of alternative invasion scenarios. Here, we ask which historical scenario best explains the colonization of Europe and Africa from South America, based on genetic data and historical modeling. In particular, we examine the number of colonization events in these regions and their timing. We also ask whether the two distinct mite lineages now in Europe coexist separately or if the current populations reflect an admixture event following colonization. To answer these questions, we first group *T. evansi* samples into clusters based on genetic variation, and then use these clusters in the subsequent analysis to infer colonization history. We argue that the ability to understand the invasion history of this species, and others like it, is critical to defining strategies for quarantine, control, and management.

## Results

### Population genetic diversity and structure

Many populations of *T. evansi* contained low levels of genetic diversity. Individuals in eight of 31 populations showed no variation across all loci (Table 1). Observed heterozygosity varied among populations from 0 to 0.288. Nine out of the 23 polymorphic populations showed significant departure from the Hardy-Weinberg genotypic proportions, resulting from a significant heterozygote deficiency. For all ABC analyses presented here, when homozygous individuals were overrepresented in a population, we down-weighted their importance by including a subset of individuals of only unique genotypes. *F*_IS_ estimates were recalculated for the data subset to verify that heterozygosity was not artificially increased, which might alter time estimates of founding events in ABC analyses.

**Table 1:** Sampling locations, geographical coordinates, host plant and number of individuals genotyped (N) by 16 microsatellites loci of the invasive spider mite *Tetranychus evansi*.

Area	Country	Locality	Genetic cluster	Lineage	Latitude	Longitude	Host plant (S*olanum spp.*)	N	*N_A_*	*A_A_*	*H_E_*	*H_O_*	*F_IS_*	*A_P_*	*N_G_*
Native	Brazil	Uruguaiana	**BR-SW**	**I**	29.77S	57.05W	*S. americanum*	34	26	1.55	0.222	0.179	0.208	3	34
Native	Brazil	Alegrete	**BR-SW**	**I**	29.78S	55.79W	*S. americanum*	28	25	1.48	0.189	0.153	0.209	1	28
Native	Brazil	Itaqui	**BR-SW**	**I**	29.13S	56.55W	*S. americanum/S. lycopersicum*	28	30	1.54	0.162	0.143	0.136	8	28
Native	Brazil	Camocim de São Félix	**BR-ATL**	**II**	8.33S	35.76W	*S. americanum*	34	19	1.1	0.026	0.013	0.418**	0	6
Native	Brazil	Goianá	**BR-ATL**	**II**	7.56S	35.01W	*S. lycopersicum*	20	17	1.02	0.003	0.003	0	0	2
Native	Brazil	Barbalha	**BR-ATL**	**II**	7.35S	39.40W	*S. americanum*	32	21	1.27	0.108	0.061	0.442**	1	15
Native	Brazil	Venâncio Aires	**BR-ATL**	**II**	29.66S	52.08W	*S. americanum*	28	16	1	0	0	-	3	1
Invaded	Kenya	Shimba Hills	**AF**	**I**	4.35S	39.31E	*S. lycopersicum*	12	17	1.03	0.005	0.005	0	1	2
Invaded	Kenya	Sindo	**AF**	**I**	0.52S	34.16E	*S. lycopersicum*	10	17	1.07	0.013	0.013	0	1	3
Invaded	Kenya	Athi-River 2	**AF**	**I**	1.39S	37.04E	*S. lycopersicum*	16	17	1.05	0.016	0.01	0.442**	0	3
Invaded	Kenya	Athi-River 3	**AF**	**I**	1.39S	37.04E	*S. nigrum*	17	17	1.04	0.01	0.004	0.652**	0	2
Invaded	Tanzania	Tengeru	**AF**	**I**	3.37S	36.80E	*S. nigrum*	21	17	1.01	0.003	0.003	0	0	3
Invaded	Tanzania	Mukuyuni	**AF**	**I**	6.55S	37.30E	*S. aethiopicum*	19	16	1	0	0	-	0	1
Invaded	Canary Is	Pooled localities^1^	**EU** 3	**I**	28 N	16 W	*S. nigrum/S. melongena/S. tuberosum/Physalis origanifolia*	12	17	1.03	0.005	0.005	0	0	2
Invaded	Portugal	Luz de Tavira	**EU** 3	**I**	37.09N	7.69W	*S. nigrum*	12	19	1.1	0.024	0.016	0.377**	0	4
Invaded	Spain	Alquerias del Niño Perdido	**EU** 3	**I**	39.90N	0.12W	*S. nigrum/S. chenopodioides*	7	16	1	0	0	-	0	1
Invaded	Spain	Valencia	**EU** 3	**I**	39.49N	0.34W	*S. nigrum*	18	16	1	0	0	-	0	1
Invaded	Spain	Benferri	**EU** 3	**I**	38.18N	0.99W	*S. nigrum*	6	17	1.01	0.003	0.003	0	0	2
Invaded	Spain	La Pobla de Vallbona	**EU** 3	**I**	39.63N	0.52W	*S. nigrum*	8	18	1.11	0.033	0.039	-0.129	0	3
Invaded	Spain	Alhama de Murcia	**EU** 3	**I**	37.82N	1.44W	*S. nigrum*	15	18	1.06	0.013	0.005	0.657**	1	3
Invaded	France	Saint-Jeannet	**EU** 3	**I**	43.74N	7.18E	*S. lycopersicum*	28	16	1	0	0	-	0	1
Invaded	Tunisia	Zaafrane	**MED**	**I**	36.19N	8.86E	*S. nigrum*	14	16	1	0	0	-	0	1
Invaded	Greece	Timpakion	**MED**	**I**	35.07N	24.77E	*S. nigrum*	16	16	1	0	0	-	0	1
Invaded	Spain	Granada	**MED**	**I**	36.72N	3.49W	*S. nigrum*	16	23	1.34	0.122	0.071	0.446	0	15
Invaded	Spain	Malaga	**MED**	**I**	36.76N	4.04W	*S. nigrum*	23	24	1.4	0.138	0.087	0.389**	0	17
Invaded	Portugal	Castanheira do Ribatejo^2^	**POR**	**II**	38.99N	8.97W	*S. nigrum*	26	32	1.92	0.321	0.191	0.422**	1	9
Invaded	Portugal	Aljustrel^2^	**POR**	**II**	37.88N	8.16W	*S. nigrum/S. lycopersicum*	27	32	1.95	0.375	0.288	0.249	0	15
Invaded	Portugal	Ameal	**POR**	**II**	40.19N	8.53W	*S. nigrum*	10	27	1.58	0.142	0.123	0.196	1	5
Invaded	Portugal	Lagos	**POR+CAT**	**II**	37.11N	8.69W	*S. nigrum*	11	16	1	0	0	-	0	1
Invaded	France	Torreilles	**POR+CAT**	**II**	42.74N	2.96W	*S. nigrum*	27	17	1.01	0.002	0.002	0	0	2
Invaded	France	Sainte-Marie	**POR+CAT**	**II**	42.71N	3.02W	*S. nigrum*	31	17	1.04	0.009	0.006	0.362**	1	3

**Figure 1:**
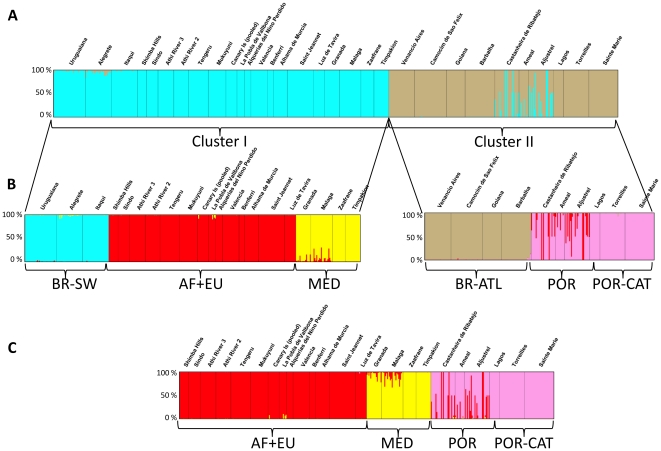
Genetic clustering of *Tetranychus evansi* samples as assessed by structure simulations using 16 microsatellite loci and displayed using distruct [**72**] for: a) entire dataset which identified cluster I and II; b) samples within cluster I and II analysed separately; c) samples from introduced areas. The associated geographical region (abbreviations) for different localities is indicated Table 1, and colour codes are as in Figure 2 and 3. Individuals are represented by vertical lines divided into parts proportional to their proposed ancestry in each structure-defined genetic cluster.

Samples are grouped by geographical regions according clustering results obtained by structure analysis (Figure 1). The belonging of samples to each of the two lineages (I and II) identified using mtDNA sequences [13] and to native and invaded areas are indicated.

*N_A_*: total number of alleles in each population, *A_A_*: allelic richness. Average expected (*H_E_*) and observed (*H_0_*) heterozygosities across loci for each population. Inbreeding coefficient (*F_IS_*) . Significant departures from HWE are given in bold: * P<0.05, ** P<0.01. Bonferroni's correction to estimate significance was used for multiple tests. *A_P_*: number of private alleles per locus per population. *N_G_*: number of multilocus genotypes.

1 Canary Is localities: Tenerife: San Miguel, La Orotavia; Gran Canaria: Santa Maria de Guia.

2 Samples including admixed cluster (I and II) individuals.

3 Corresponding to Europe excluding MED localities, plus Canary Is.

Previous work by Boubou *et al.* [13] based on mitochondrial *COI*, found evidence for two groups of mites (lineages I and II) with divergent evolutionary histories. Here, we first tested whether variation in nuclear loci also supported these clusters, and then we used the results in the subsequent ABC analyses. A Bayesian clustering analysis of microsatellite data implemented in structure confirmed the presence of two genetic clusters, I and II (Figure 1a), which corresponded perfectly to the previously recognized *COI* lineages [13]. The total likelihood increased with increasing K proposed clusters, although the likelihood began to plateau after K = 5. In examining ΔK for K = 1–11 a single distinct peak emerged at K = 2, a result found in 10 runs performed. Although most individuals were unambiguously assigned to either of the two clusters (posterior probability *>*90%), a few individuals originating from two Portuguese populations (3 and 2 individuals from Castanheira do Ribatejo and Aljustrel, respectively) were assigned to cluster I with a high posterior probability (*>*98%), while the majority of Portuguese mites fell into cluster II. In addition, some individuals from these two populations (4 and 5 in Castanheira do Ribatejo and Aljustrel, respectively) and from Ameal (2 individuals) were identified as possessing apparent hybrid ancestry between the two clusters (approximately 50% assignment to each cluster, Figure 1b). The existence of two major clusters is also supported by AMOVA analyses, in which 59% of the genetic variation was explained by genetic differentiation between cluster I and II (Table 2a). When AMOVA was used to test for geographic structure within and between native and introduced areas, differences between populations within each of the geographical ranges (native and introduced areas) contributed to 62% of total variance, while only 27% was attributed to the variation between the ranges (Table 2b). The occurrence of individuals associated with clusters I and II in both native and introduced (Europe) areas accounts for this pattern.

**Table 2:** Hierarchical analysis of molecular variance (AMOVA) showing the partition of the genetic variation among populations of *Tetranychus evansi* from: a) two major clusters (I and II) identified by structure (see Figure 1); b) native and introduced areas (see Table 1 for localities included in each area).

Source of variation	d.f.*	Sum of squares	Variance components	Percentage of variation	*P*-value
a) Cluster I and II					
Among clusters	1	2181.298	3.42176	59.32	<0.001
Among populations within clusters	31	2217.926	1.83050	31.73	<0.001
Within populations	1265	652.668	0.51594	8.94	<0.001
b) Native and introduced areas					
Among areas	1	877.730	1.28990	27.25	<0.001
Among populations within areas	31	3521.513	2.92436	61.84	<0.001
Within populations	1265	652.668	0.51594	10.91	<0.01

Probability test *P*-value calculated by 1000 permutations.

* Degrees of freedom (d.f.).

When structure was run with populations from cluster I only, three highly significant clusters (K = 3) were identified (Figure 1b) corresponding to samples from the following areas: BR-SW: southwest Brazil (Uruguaiana, Alegrete and Itaqui); MED: Mediterranean localities [southern Spain (Granada and Malaga), Greece and Tunisia] and AF+EU: eastern Africa (AF) plus European localities (EU) apart from those included in MED (Table 1). An additional cluster emerged when populations of cluster II were analysed separately (Figure 1b), essentially distinguishing admixed individuals in Portugal. When a finer structure was explored for individuals from the invaded area alone, the three clusters previously identified clearly emerged: AF+EU, MED and POR-CAT (Figure 1c). The same general pattern of genetic structure obtained with structure was also supported by Principal Component Analysis (data not shown).

Most of the introduced populations in cluster II displayed low genetic polymorphism (Table 1) and weak or insignificant genetic differentiation (mean pairwise *F*_ST_ = 0.13; data not shown), hence further tests of alternative introduction scenarios were not performed with this cluster. For populations in cluster I, alternative invasion pathways were exhaustively compared in a subsequent ABC analysis.

### Inference of introduction scenarios and estimation of historical parameters

**Figure 2:**
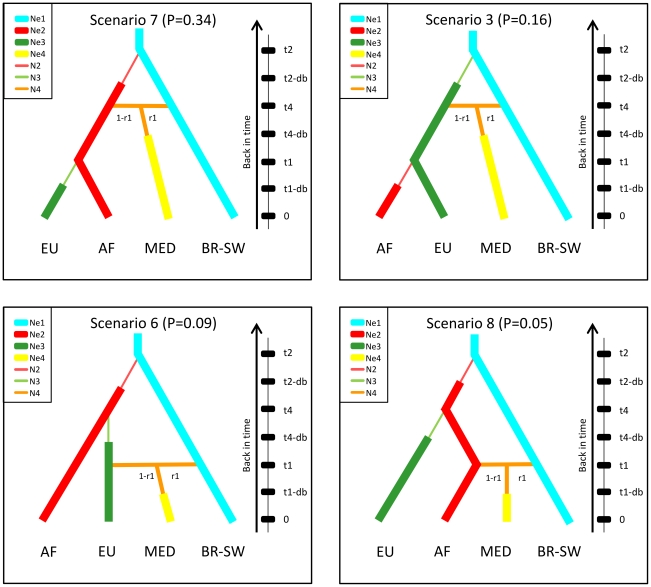
Schematic representation of four competing scenarios considered for the introduction routes of *Tetranychus evansi* in Africa and Europe tested by Approximate Bayesian Computation (ABC) analysis. Four populations were considered in each analysis as identified by the clustering structure analysis (see text and Figure 1). Ne*i* and N*i* correspond to effective population sizes and number of founder individuals, respectively, and they were assumed to be different in all considered populations. The time of event (*ti*), in number of generations, corresponds to the time at which an introduced population has diverged from its source population; the duration of the initial bottleneck (*db*) was assumed to be the same in all introduced populations. Time 0 is the sampling date. Admixture rate *r* relative to population BR-SW and 1-*r* to either population AF (scenarios 7 and 8) or EU (scenarios 3 and 6). We assumed that all populations evolved as isolated demes and no exchange of migrants occurred after the introduction. All parameters with associated prior distributions are described in Table S2.

In all historical scenarios tested the BR-SW population was always considered as ancestral to all other populations. When comparing all of the 24 competing scenarios (Figure S1) two features of the *T. evansi* invasion emerged. First, the colonization of the African continent (AF) by the Brazilian population (BR-SW) preceded the arrival of the mite in Europe (EU). This hypothesis is supported by five scenarios, which result in a cumulative posterior probability of 0.66. By contrast, the cumulative probability of the four scenarios supporting the colonization of Europe before that of Africa is 0.31 and the scenario of the invasion of the MED population before the others was very poorly supported (posterior probability 0.03). Second, the MED population resulted from an admixture event having occurred between the African (AF) population, and a second independent introduction of the same Brazilian (BR-SW) origin. The choice of this admixture event is supported (*p*<5%) in six scenarios (cumulative posterior probability 0.71). Among the four best supported scenarios of an admixed MED population (Figure 2), scenario 7 has the highest posterior probability (0.34) with an admixture between AF and BR-SW mites estimated at 34.4% and 66.6% respectively (*r1* = 34.4, 90% CI: [25.6–82.0%]). The admixture between the same populations also appears in scenario 8 (posterior probability 0.05), except that the admixture event is estimated to have occurred later, posterior to the mite entry in Europe. Likewise, two other scenarios (scenarios 2 and 4, Figure S1) out of the 6 supporting an admixture in the MED populations estimate the entry of the mite in EU much earlier than in the AF and MED regions (posterior probabilities <0.05; data not shown). Finally, in scenarios 3 and 6 (respectively 0.16 and 0.09 posterior probabilities) the admixed MED population derived from the EU and the BR-SW populations (Figure 2). Genetic diversity in EU is reduced compared to MED (*A_A_*: 1.040 and 1.185 respectively; Table 1) which further favors an older origin of the latter. Likewise the hypothesis of the mite arriving first in Europe is not supported by available historical records from these continents as discussed below.

**Figure 3:**
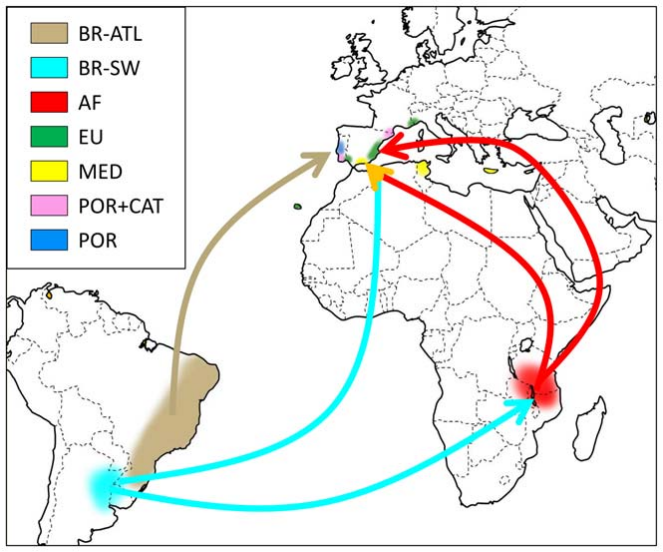
Most likely scenario of invasion describing the routes of introduction of *Tetranychus evansi* from South America into Africa and Europe. The arrows indicate the most likely pathway. Populations were defined according to structure analysis (Figure 1). See Figures 1 and 2 for color codes and Table 1 for population abbreviations.

**Figure 4:**
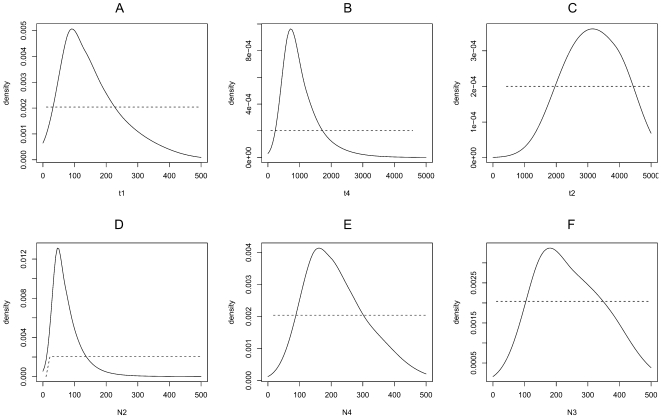
Prior and posterior distributions density curves for different estimators of *Tetranychus evansi* introduction events, calculated under scenario 7 (the most likely scenario of invasion); a) *t1*: the divergence time between eastern African (AF) and European (EU) populations; b) *t4:* between Mediterranean (MED) and eastern African (AF) populations; c) *t2*: between (BR-SW) and (AF) populations; d) N2; e) N4 and f) N3: estimates of the propagule size for introductions in AF, MED and EU respectively. Geographical codes as in Table 1. Y-axis: probability density of estimated parameters. The dotted and solid lines correspond to the prior and posterior density curves, respectively. The best estimates of parameters occur where the posterior probability density function peaks.

The most likely scenario of invasion deduced from analyses based on ABC computation is summarized in Figure 3. Under this scenario, mites from lineage I were introduced to Africa (AF) from southwest Brazil (BR-SW). From there two pathways of introduction into Europe occurred, a first leading to the EU population and a second involving the admixture event with an additional introduction from the same BR-SW origin leading to the MED admixed population. Several historical parameters help to further define this scenario. Differences between the posterior and prior density curves for times of introduction events (*t1* and *t4*) were well marked, with clearly peaked posteriors (Figure 4), indicating that the genetic data are informative for these parameters. The admixture event between AF and BR-SW leading to the MED population was estimated at 872 generations ago (*t4*), which roughly corresponds to 72 years, assuming 10 (87 years) to 15 (58 years) generations of *T. evansi* per year in the Mediterranean-invaded areas (estimates modelled with Climex; parameters from [15]; Migeon, unpublished data). The posterior distributions support a small divergence time (*t1*) between AF and EU populations (median = 132 generations (90% CI: [33.4–363], i.e. 11 [13]–[9] years). While being a rough estimate, it clearly dates the introduction of *T. evansi* in Africa long before the arrival of the mite in Europe, which is consistent with first records of the mite in this area (EU), in particular considering the small upper confidence limit. Estimates of the introduction time of the mite in Africa remains however poorly defined (*t*_2_) with a median of 3200 generations ago (90% CI: [1570–4750]). According to scenario 7 (the most likely one), the divergence between the Brazilian (BR-SW) and the African (AF) populations was dated (*t*_1_) at 133 [106–160] years ago, assuming 20 to 30 generations per year in tropical Africa (Migeon, unpublished). However, because of the scenario choice, the time period involved in the *t*_2_ simulation is much longer than for *t*_1_; the probability to estimate accurately short time periods is superior. Differences between the posterior and prior density curves for population size associated to the introduction events suggest that the invasion resulted in a reduced number of mites having arrived in AF (*N*2 = 61), in MED (*N*4 = 202) and in EU (*N*3 = 231) (Figure 4). While the information provided remains limited, the three events are rather similar supporting the hypothesis that a small number of migrants colonized the new geographical regions.

### Test of admixture in Portugal and timing

**Figure 5:**
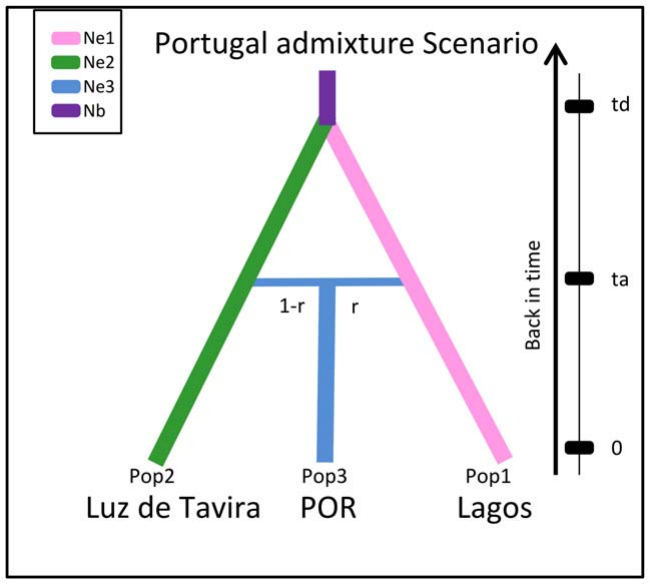
Schematic representation of the admixture scenario tested in ABC analysis of populations in Portugal. Two parental populations studied: Luz de Tavira and Lagos (corresponding respectively to AF+EU and POR-CAT clusters as in Figure 1) with constant effective population sizes *N*e1 and *N*e2, having diverged at time (*td*) from an ancestral population of size *N*b. At time *ta*, an admixture event occurred between individuals from Lagos (1) and Luz de Tavira (2) giving birth to an admixed population POR (3) with effective size *N*e3 and with an admixture rate *r* relative to population 1 and 1-*r* to population 2. All parameters with associated prior distributions are described in Table S2.

**Figure 6:**
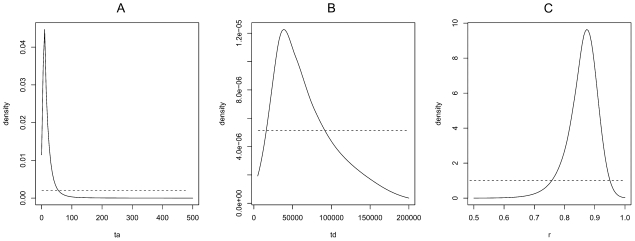
Prior and posterior distributions density curves calculated under an admixture scenario of *Tetranychus evansi* introduced populations in Portugal, with parameters: a) *ta:* the admixture time in the hybrid population (POR), b) *td*: the divergence time between the two parental populations (Luz de Tavira and Lagos) belonging respectively to the two main clusters (I and II) detected by structure analysis (Figure 1), and c) *r*: genetic admixture rate. The dotted and solid lines correspond to the prior and posterior density curves, respectively. Y-axis: probability density of estimated parameters. The best estimates of parameters occur where the posterior probability density function peaks.

The hypothesis of a hybrid origin of part of the Portuguese population was also clearly supported by the ABC analysis (Figure 5). Demographic parameters estimated under this scenario are presented Figure 6. Peaked posteriors were marked for time events (*td* and *ta*: divergence and admixture times, respectively) and the admixture rate (*r*). The divergence between the two parental populations was dated at a median of 56,188 generations ago (approximately 2,341 [2,809 –1,873] years ago), whereas admixture time (*ta*) was estimated at only 13 generations. The admixed Portuguese population results from mites from Lagos and Luz de Tavira in estimated proportions of 0.87 and 0.13 respectively.

## Discussion

### Genetic inferences and multiple introductions

Genetic signatures from multi-locus microsatellites clearly assigned invasive populations of *T. evansi* to two well-supported clusters, which matched the *COI* lineages I and II found previously by Boubou *et al* [13]. Despite the high level of divergence between these two lineages (2.84% based on *COI* sequences) and the old divergence time between them (estimated at about 5,000 years based on mitochondrial data), it is clear from the admixture event detected in Portugal and also by previously identified heterozygote ITS sequences [13], that crosses between the two clades do occur in nature. Additional evidence that the clades are indeed one species is provided by cross breeding experiments in the laboratory between mites from the two haplotypes, I and II [28]. The admixture detected in Portugal demonstrates not only that individuals from different native-range sources coexist but also that interbreeding between invasive populations occurs in nature. Yet, the signature of admixture was only found in a very restricted geographic area, raising the question as to why the 2 haplotypes do not form a more widespread panmictic population. There are at least two (non-mutually exclusive) hypotheses to explain this pattern: 1) the occurrence of both haplotypes in the same geographical location is a recent event, hence there has not been enough time for mixing between populations to occur; 2) there are intrinsic and/or ecological incompatibilities between haplotypes. The partial incompatibility found by Gotoh *et al* [28] lends support to the second hypothesis. In the current analysis, we can reject the hypothesis of a single introduction of *T. evansi* in Europe. Rather, this invasion resulted from at least three independent introductions. Two originated from highly divergent sources in Brazil, lineage I and II. A third introduction involved lineage I also from Brazil, but arriving at Europe through Africa.

### Invasion pathways and admixture events

As predicted by the hypothesis of South America being the native area for this mite, higher genetic diversity was found in Brazil compared to the introduced range, except the admixed populations from Portugal. However, a precise source of the invasion could not be assigned. This lack of resolution might result from under-sampling source regions which can lead to underestimating native diversity [29]. *Tetranychus evansi* persists at very low densities in Brazil, where it is not considered a pest, as in most of South America [30], making it difficult to find the mite in the field. Consistent with this observation and adding further evidence for a neotropical origin of the species, natural enemies control *T. evansi* densities in these regions, as expected in co-evolving prey-predator systems [31].

In its introduced range, *T. evansi* was first reported in Africa, with records from Mauritius in mid-1950's, La Réunion, 1976, Zimbabwe, 1979, South Africa, 1985, Kenya, 2001 and Tanzania, 2003 (see [15] for the known world distribution). In all cases, these records are associated with outbreaks of the mite. The connection of historical records and pest outbreaks also applies to Europe, with occurrences recorded from 1991 in Portugal, 1995 in Spain, 2004 in France, 2005 in Italy and 2006 in Greece [32], [33], [34], [35], [36]. Because historical data indicates that Europe (EU) and Africa (AF) were invaded recently and because they are included in the same structure cluster (Figure 1c), we tested whether AF or EU is the potential source of MED, or whether the opposite scenario holds i.e. [AF→MED] or [EU→MED]. Our results support that the mite first arrived in Africa and only later in Europe.

An additional level of complexity of the invasion as inferred from ABC, could be detected in southern Spain (MED) where the mites resulted from an admixture of mites from Africa (AF) and an independent introduction from southwest Brazil (BS-SW). Consistently, the MED group includes the most polymorphic populations (Malaga and Granada) in the worldwide invaded areas (apart from Portugal where mites resulted from an admixture of the two divergent I and II lineages). Genetic data suggest the arrival of *T. evansi* in the Mediterranean region before historical records (approximately 70 years ago), so that a time lag might have occurred before the mite was first reported from southern Spain in mid-1990. This discrepancy could be due to the fact that, although high agricultural activity in southern Spain has been accompanied by intensive pest detection programs (F.J. Ferragut Pérez, pers. com.), it is likely that spider mites can remain hidden in surrounding vegetation until they switch to crops and reach pest levels. For example, the palm mite, *Oligonychus afrasiaticus*, was first reported in Israel early 1980's on weeds, but only started to cause commercial damage on palm trees almost two decades later [37].

In the case of eastern Africa, estimates of the mite introduction in the continent remains poorly defined. According to ABC analysis, the Brazilian (BR-SW) and the African populations diverged about 130 years ago. Yet, according to historical records, the mite arrived much more recently in Africa (Zimbabwe in 1979 [38]) even counting the first report in the area, which is from Mauritius and goes back to mid-1950's [16]. An overestimation of the divergence time can be explained by the fact that the population sampled in Brazil is not the actual Brazilian ancestor but a related population from which the mites were collected in BR-SW diverged and were used in ABC analyses. Also, as for the colonization of Europe, a lag-time between the introduction and the detection of the mite could have occurred, with the mite remaining undetected for long time. As for many Tetranychidae, identification requires the expertise of skilled taxonomists [19] and the species can be easily confounded with other closely related spider mites, as happened in Japan [28] and in an early report of *T. evansi* from Brazil, Bahia, where it was mistakenly under the name of *Tetranychus marianae* [39].

Setting aside precise dates of the introductions, contemporary genetic data support the invasion scenario of introduction of the mite from southwest Brazil (BR-SW) to eastern Africa (AF), which was the source of both the admixed MED population from southern Spain and other European populations (EU), the later displaying an expanded distribution, having been recently detected in remote areas in east Asia, as in Japan and China. This colonization scenario is consistent with an invasive “bridgehead” effect, in which a particular invasive population serves as the source of colonists for other areas [26]. The “bridgehead” pattern shares some similarities with the “long-distance jump dispersal” pattern, in that invasive populations stem from previous invasions [27].

The analysis supports a second independent source of introduction from the Brazilian atlantic coast (BR-AT). These populations (cluster II) exhibited low genetic diversity limiting our ability to test alternative invasion pathways. However in the invaded region, mites of cluster II were found widely in Portugal, but very restricted in Catalonia, despite intensive surveys done in Southern Europe. We did not have the statistical power to confirm the scenario of mites arriving directly from Brazil to Portugal; however this pathway was better supported than the alternative scenario of mites arriving from Brazil to Catalonia and from there to Portugal.

The geographical distributions of the two distinct invading genotypes, lineages I and II, suggest that the two mite lineages differ in invasive potential. The occurrence of two invasion biologies or syndromes in *T. evansi* thus provides an excellent opportunity to evaluate what biological traits differ among invasive or non-invasive populations.

### Genetic variation and invasiveness

The introduction of species in new environments is often associated with founder events resulting in low genetic variability and consequently low evolutionary potential. Nevertheless, numerous invasions have successfully occurred despite these genetic bottlenecks [40], [41], [42], [43], [44]. However, recent reviews have suggested that many successful invading populations have not experienced significant loss of genetic diversity relative to native populations [45], [46], [47], [48]. One common hypothesis used to explain this is that the invasive populations may stem from multiple introductions [45], [49]. The colonization of new regions by individuals from genetically disparate native sources has the potential to increase genetic variation by interbreeding and offset genetic bottlenecks [50], [51], [52]. Rapid population growth after colonization can also limit the loss of genetic variation in founder events [53].

In the case of *T. evansi*, introduced populations were found to exhibit low genetic polymorphism and in some cases they were invariable, raising the apparent paradox of how they become successful invaders despite experiencing significant genetic bottlenecks during colonization [54]. Nevertheless, given that Europe was colonized by mites with three different origins (two with lineage I and one with lineage II, albeit the latter has been less successful than the formers), admixture may occur between populations from these different routes. According to our analysis, two admixture events were found, and these could lead to an increase in the genetic variability of invading populations. In Portugal admixture has occurred between the two lineages (I and II) of *T. evansi*. It remains to be tested if new recombinant invasive genotypes via intraspecific hybridization resulting from admixture of the two lineages possess novel phenotypes that could facilitate invasion success. Such genetic changes can be substantial and might have unexpected behavioural or physiological consequences [55]. Given that there are currently populations from the three invasion routes still present in Europe, one may wonder whether more admixture events will occur among populations from different routes, or if mites from one invasion route will eventually take over the whole continent.

Research has also suggested that adaptive evolution may play a crucial role in invasions of some species [56]. Considering that *T. evansi* is thought to be constrained in its range by the absence of diapause in colder climates [15] and by its ability to exploit novel host plants, understanding biological traits that are crucial for the success of the invasion of *T. evansi* in more temperate climates is sorely needed. For an agricultural pest such as *T. evansi*, dissecting biological traits favoring the invasion potential will also provide relevant information to management strategies in the face of global change [57].

### Approximate Bayesian Computation (ABC) and understanding complex invasions

This study confirms that recent advances in approximate ABC approaches to reconstructing historical demography can be particularly informative for the study of invasive species, even when invasion histories are complicated. A great number of alternate scenarios can often be constructed to explain current invasions. Here we challenged the genetic data with 24 possible invasion histories (as described in the Material and Methods section), and were able to unravel a rather complex invasion history. The methods employed were able to reconstruct two independent and complex pathways of recent invasions, in spite of the incomplete nature of historical collections, difficult taxonomy, and low levels of genetic variation at multiple nuclear loci. The method also raises the issue of lag-times between the detection of the mite documented by field records and its actual introduction in a new area, a phenomenon that has been problematic for both invasion biologists and managers [46], [47], [58]. Obviously, ABC methods also suffer from shortcomings. In particular, and given the intensity of trading, travelling, and hence the opportunities for inadvertent invasions to occur, invasion scenarios may become increasingly complex, as more multiple invasions can occur. Therefore, it is always conceivable that a more complex, but historically sound, invasion scenario has not been included in the analysis performed.

While ABC methods could only partially resolve the most recent events for which minimal genetic variation exists, it is likely that this limitation will be short-lived however as “next-generation sequencing” approaches provide the promise of nearly unlimited numbers of loci for population studies [59]. With the recent availability of the whole genome sequence of the spider mite *Tetranychus urticae* [60], a new flood of genome-wide markers (e.g. single nucleotide polymorphism, [61]) and/or fully sequenced population samples of genomes will become accessible for this group of mite pests. The results also suggest that ABC methods will be useful as part of on-going programs of biosecurity and pest management [62], largely because of the ability to (1) distinguish among alternative scenarios of invasion pathways, and (2) estimate temporal components of invasions, including pre-detection lag-times.

Accumulating studies of biological invasions reveal that invasions can be much more complex than they appear, as a result of multiple, cryptic invasion events and pathways, the potential for past and recent admixture, among other factors [63]. *Tetranychus evansi* is in some ways a worst-case scenario for testing these approaches, in that the species is small with little available morphological variation, and historical records are incomplete and misleading. Here, we demonstrate how ABC can provide a framework within which to test alternative hypotheses concerning historical parameters for complex biological invasions, such as that of the mite *T. evansi*.

## Materials and Methods

### Population samples and markers

Individual mites were collected from a total of 31 localities representing both native (Brazil) and introduced (Africa and Europe) areas (Table 1). A total of 606 individuals were genotyped for 16 microsatellite loci previously isolated [64] and used in two multiplex PCR sets as described in Table S1. Either the forward or reverse primer for each locus was 5′end labelled with a fluorescent dye (FAM, NED, PET or VIC). Total DNA was extracted from single mite female using the DNeasy® Blood & Tissue Kit (Qiagen) following the spin-column protocol from Cultured Animal Cells slightly adapted for mites, dividing volumes by two, with incubation at 70°C for 3 hours and a final elution in 50 µL of DNase-free water. Amplifications were performed using the QIAGEN® Multiplex PCR Kit. Multiplex PCRs were conducted in 10 µL reaction volume containing 1× QIAGEN® Multiplex PCR Master Mix (including HotStarTaq DNA Polymerase, dNTPs and 3 mM of MgCl_2_ as final concentration), about 10 ng of total DNA and DNase-free water. Multiplex PCRs consisted of an initial denaturation at 95°C for 15 min, 40 cycles at 94°C for 30 sec, annealing at 57°C for 90 sec, extension at 72°C for 60 sec and a final extension of 60°C for 30 min. PCR products were detected using an ABI PRISM 3130xl (Applied Biosystems), 1 µL of the PCR product diluted at 1/300 was mixed with 18.9 µL of Hi-Di™ formamide (Applied Biosystems) and 0.1 µL of GeneScan™ −500 LIZ® Size Standard (Applied Biosystems). Alleles were scored using GeneMapper® V4.0 (Applied Biosystems).

Sequences of the ITS region (1,137 bp) and a portion of the mt *COI* gene (868 bp) previously obtained for most of the genotyped mite individuals was used to assign samples to either lineage I or II previously described in [13].

### Population structure

Indices of genetic diversity (Table 1) were calculated using dnasp v.4.20.2 [65]. The genetic structure of all native and introduced populations was estimated by pairwise *F*_ST_ computed under the Kimura 2 parameter (K2P) model [66] using arlequin version 3.1 [67]. The partitioning of genetic variance was investigated by using molecular analysis of variance AMOVA [68] as implemented in arlequin. Two sets of groups were compared: 1) populations within two clusters (I and II; detected by the program structure as described below); and 2) populations within native and introduced areas. Permutations to determine significance were set at 10,000 and the distance method used to determine variation in the dataset was the number of different alleles present in each group.

The program structure v.2.3.3 [69], [70], which implements a model-based clustering method, was used to infer the number of genetic clusters (K = number of clusters of individuals characterized by allele frequencies at each locus) present in the dataset, and to assign individuals to these clusters. We used the admixture model with correlated allele frequencies and did not include any *a priori* information about collection sites. In all simulations performed in structure, we used a “burn-in” period of 5×10^4^ followed by a run length of 5×10^5^ MCMC iterations, which were sufficient to give a stable α and estimate of the log probability of the data. We first ran separate models including the whole data with K ranging from 1 to 12. We selected the upper K value as 12, based on the number of groups of populations identified by *F*_ST_ values (data not shown). Because two main clusters were identified (I and II), these were submitted separately to a second run of analyses, using K = 1–5 and K = 1–7, respectively. Finally, a third analysis was performed on all introduced populations (K = 1–5) from Europe and Africa. For each value of K, 10 independent runs were computed. To estimate the most likely K value, we used the ΔK statistics [71] in structure. Results are displayed using distruct program [72].

Because some of the assumptions of structure, e.g. as Hardy-Weinberg (HW) equilibrium within populations and linkage equilibrium, might be violated, in particular HW for arrhenotokous species, as often occurs for spider mites [73], population structure was also examined by an individual based Principal Component Analysis implemented in the program pca-gen version 2.9.3 [74].

### Inferring colonization scenarios using Approximate Bayesian Computation

Approximate Bayesian Computation (ABC) analysis was performed using samples in the cluster I identified by structure, further refined based on diversity indices information (Table 1). The native cluster (BR-SW) gathered three southern Brazilian samples from Uruguaiana, Alegrete and Itaqui, however, the last population was eliminated from ABC analyses because it had the highest proportion of private alleles at high frequency and shared only 29% of their alleles with introduced populations (data not shown). Although eastern African (AF) and European (EU) samples showed a high genetic homogeneity and were gathered in a sub-cluster (Figure 1b), they were separated for ABC analysis into two populations: eastern African (AF) and Europe (EU) to test their invasion trajectories independently. In the genetic cluster MED (Table 1, Figure 1b), only the Spanish samples, Granada and Malaga, were considered because the two Tunisian and Greek populations were monomorphic at all loci and their shared single multi-locus genotype was present in these southern Spanish samples.

Tentatively all possible evolutionary scenarios leading to the following four populations were included in the analysis with the only two conditions: i) the BR-SW population was considered as ancestral to all other populations and ii) the MED population was the only population potentially issued from an admixture of BR-SW and either one of the two remaining populations. A total of 24 scenarios (Figure S1) were found to comply with these two conditions and were all included in the ABC analysis. We generated a reference table containing 24×10^6^ simulated data sets (on average 10^6^ per scenario), each scenario being given a uniform prior probability (1/24). The prior distributions of parameters are reported Table S2. The estimation of posterior probabilities of each scenario was performed through a polychotomous logistic regression on the 10% simulated data sets closest to the observed one in the space of normalized summary statistics, each simulated data set being weighted by its Euclidian distance through an Epanechnikov kernel [75]. Posterior distribution of historico-demographic parameters followed the ABC method described in [76], taking the 10,000 (1%) simulated datasets closest to the observed dataset for the local linear regression after applying a *logit* transformation to parameter values. All ABC computations were performed using a beta-version of the program diyabc v.2 [77].

### Parameters of the admixture event in Portugal

The occurrence of an admixture event in Portugal, suggested by microsatellite data in mites collected from this region, was tested using ABC methods. One admixed population, formed from pooling the two Portuguese populations (Castanheira do Ribatejo and Aljustrel) and two parental populations (Luz de Tavira and Lagos), was created based on information obtained from the structure analysis (see Results section and Figure 1b). The proportion (*r*) of alleles of the parental population Lagos in the admixed population and the time of the divergence (*td*) and the admixture events (*ta*) were estimated according to the evolutionary scenario shown in Figure 5. Prior distributions of these three parameters are shown Figure 6. As above, a reference table of 1.5×10^6^ simulated data sets was generated and posterior distributions of parameters were estimated using the 15,000 (1%) simulated datasets closest to the observed dataset for the local linear regression, after application of a *logit* transformation to parameter values. diyabc was used to estimate all ABC computations.

## Supporting Information

Figure S1 **Schematic representation of the 24 competing introduction scenarios considered for the inference of the introduction routes of** ***Tetranychus evansi*** **in Africa and Europe tested by ABC analysis.** Four populations were considered in each analysis as identified by the clustering structure analysis (see text and Figure 1): Pop1 – BR-SW is the native population from southwest Brazil, Pop2 – AF corresponds to African samples, Pop3 – EU corresponds to European samples *pro parte*, Pop4 – MED corresponds to Mediterranean samples (Andalusia in southern Spain, Tunisia and Crete). N2, N3, N4 correspond to number of founder individuals and were assumed to be different in all introduced populations. NB, NA, NM, NS correspond to stable effective population size in Pop1 – Br-SW, Pop2 – AF, Pop3 – EU and Pop4 – MED, respectively. The time of event (*ti*), in number of generations, corresponds to the time at which an introduced population has diverged from its source population; the duration of the initial bottleneck (*db*) was assumed to be the same in all the introduced populations. Time 0 is the sampling date. Admixture rate *r* relative to population Pop1 – BR-SW and 1-*r* to either population Pop 2 – AF or Pop3 – EU. We assumed that all populations evolved as isolated demes and no exchange of migrants occurred after the introduction. All parameters with associated prior distributions are described in Table S2.
(TIF)

Table S1 **Microsatellite loci assembled in two multiplex PCR sets, used to genotype** ***Tetranychus evansi*** **mites (details on microsatellite isolation and characterization are described in** [**64**] **).** The fluorescent dye (FAM, NED, PET or VIC) used for each locus to 5′end label either the forward or reverse primer is indicated.
(XLS)

Table S2 **Prior distribution of demographical and historical parameters describing: a) the 24 introduction scenarios of** ***Tetranychus evansi*** **in the Europe and Africa and b) in the admixture model in Portugal.** Prior distributions for marker parameters were the same in the two ABC analyses.
(XLS)
