# Supplementary figures and images for: Test of Colonisation Scenarios Reveals Complex Invasion History of the Red Tomato Spider Mite Tetranychus evansi
Source: PLoS One. 2012 Apr 23;7(4):e35601. doi: 10.1371/journal.pone.0035601 (PMC3335100; doi:10.1371/journal.pone.0035601)

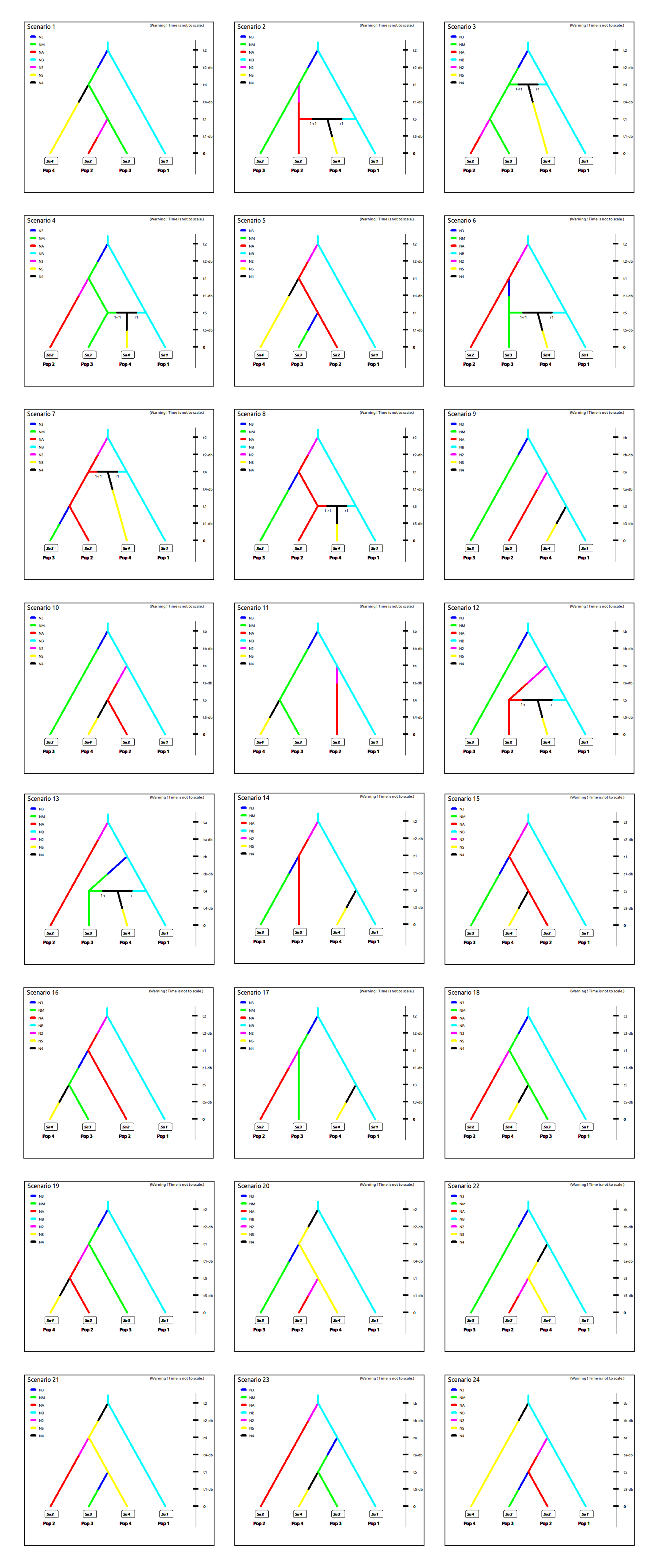

Supplement: Figure S1 — Schematic representation of the 24 competing introduction scenarios considered for the inference of the introduction routes of Tetranychus evansi in Africa and Europe tested by ABC analysis. Four populations were considered in each analysis as identified by the clustering structure analysis (see text and Figure 1): Pop1 – BR-SW is the native population from southwest Brazil, Pop2 – AF corresponds to African samples, Pop3 – EU corresponds to European samples pro parte, Pop4 – MED corresponds to Mediterranean samples (Andalusia in southern Spain, Tunisia and Crete). N2, N3, N4 correspond to number of founder individuals and were assumed to be different in all introduced populations. NB, NA, NM, NS correspond to stable effective population size in Pop1 – Br-SW, Pop2 – AF, Pop3 – EU and Pop4 – MED, respectively. The time of event (ti), in number of generations, corresponds to the time at which an introduced population has diverged from its source population; the duration of the initial bottleneck (db) was assumed to be the same in all the introduced populations. Time 0 is the sampling date. Admixture rate r relative to population Pop1 – BR-SW and 1-r to either population Pop 2 – AF or Pop3 – EU. We assumed that all populations evolved as isolated demes and no exchange of migrants occurred after the introduction. All parameters with associated prior distributions are described in Table S2. (TIF) [file pone.0035601.s001.tif]
